# Abfall von Sauerstoffsättigung und Blutdruck sowie Anstieg des zentralen Venendrucks im Rahmen eines Mitralklappenclippings bei einer 81-Jährigen

**DOI:** 10.1007/s00108-021-01241-4

**Published:** 2022-02-07

**Authors:** Martin J. Volz, Matthias Aurich, Mathias Konstandin, Hugo A. Katus, Norbert Frey, Michael M. Kreusser, Philip W. Raake

**Affiliations:** 1 Klinik für Innere Medizin III, Abteilung für Kardiologie, Universitätsklinikum Heidelberg, Im Neuenheimer Feld 410, 69120 Heidelberg, Deutschland; 2 DZHK-Standort Heidelberg/Mannheim, Heidelberg/Mannheim, Deutschland

**Keywords:** Mitralklappeninsuffizienz, Mitralklappenrekonstruktion/Komplikationen, Hämodynamische Komplikationen, Iatrogener Atriumseptumdefekt, Vorhofseptumokkluder, Mitral valve insufficiency, Mitral valve repair/complications, Hemodynamic complications, Heart septal defects, atrial/iatrogenic, Septal occluder device, atrial

## Abstract

**Hintergrund:**

Atriumseptumdefekte (ASD) im Rahmen eines endovaskulären Mitralklappenclippings sind potenziell hämodynamisch relevante Komplikationen. Ein sofortiger Verschluss mittels Okkluder kann eine sichere und effektive Therapie darstellen.

**Fallzusammenfassung:**

Eine 81-jährige Patientin mit schwerer Dyspnoe bei vorbekannter hochgradiger Mitralklappeninsuffizienz wurde zum elektiven Mitralklappenclipping vorgestellt. Die Clipimplantation verlief komplikationslos. Nach Entfernung der transseptalen Schleuse kam es zu einem plötzlichen Abfall der Sauerstoffsättigung und des Blutdrucks sowie zu einem sofortigen Anstieg des zentralen Venendrucks. Es zeigte sich ein iatrogener Links-rechts-Shunt auf Vorhofebene mit relevantem Shuntvolumen. Daraufhin erfolgte der sofortige Verschluss mittels Vorhofseptumokkluder, was zu einer unmittelbaren Besserung der Kreislaufparameter und der Sauerstoffsättigung führte.

**Schlussfolgerung:**

Ein Anstieg des zentralen Venendrucks, ein Blutdruckabfall oder Sättigungsabfall nach Rückzug der transseptalen steuerbaren Schleuse im Rahmen des Mitralklappenclippings sollte bezüglich eines möglichen ASD abgeklärt werden.

## Abstract (en)

**Background:**

Atrial septal defects (ASD) following endovascular mitral valve clipping are potentially hemodynamically relevant complications. Immediate closure with an occluder can represent a safe and effective treatment.

**Case summary:**

An 81-year-old female patient suffering from severe dyspnea due to previously known severe mitral valve regurgitation was scheduled for elective mitral valve clipping. The clip was successfully implanted. Removal of the transseptal cannula resulted in a sudden drop in oxygen saturation and systolic blood pressure as well as an immediate increase in central venous pressure. An iatrogenic left-right shunt was observed at the atrial level with a relevant shunt volume. Immediate closure using an atrial septal occluder successfully restored the oxygen saturation and hemodynamic parameters.

**Conclusion:**

An increase in central venous pressure, reduction of systolic blood pressure or oxygen saturation after withdrawal of the transseptal cannula during mitral valve clipping should always be further investigated regarding a possible ASD.

## Anamnese

Eine 81 Jahre alte Patientin stellte sich in unserer Abteilung zum elektiven Mitralklappenclipping bei hochgradiger Mitralklappeninsuffizienz aufgrund eines partiellen Sehnenfadenabrisses vor. Die Patientin war bereits zuvor bei hochgradiger Aortenklappenstenose mit einer transarteriellen Aortenklappenprothese versorgt worden. Postprozedural blieb jedoch eine Belastungsdyspnoe bei leichter Belastung bestehen. Des Weiteren lagen eine bekannte koronare 3‑Gefäß-Erkrankung, paroxysmales Vorhofflimmern, eine noch nicht weiter abgeklärte Struma multinodosa, eine Gichtarthropathie sowie ein kompletter Linksschenkelblock bei Aufnahme vor. Aufgrund von persistierender Belastungsdyspnoe, peripheren Ödemen sowie deutlich erhöhtem Operationsrisiko hinsichtlich eines herzchirurgischen Eingriffs wurde beschlossen, eine endovaskuläre Mitralklappenrekonstruktion mittels Clip durchzuführen.

## Diagnostik

**Figure 1:**
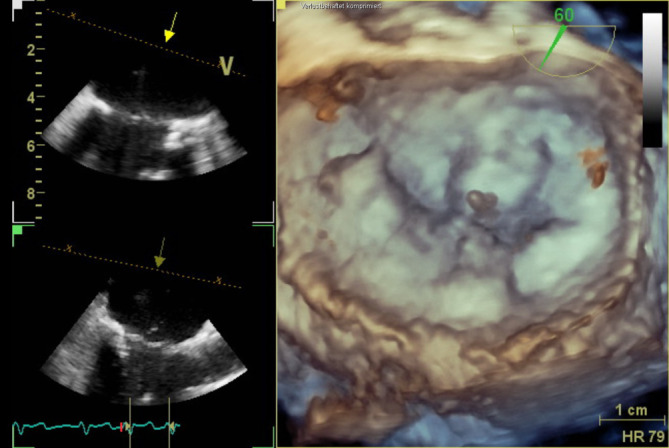

In der *transösophagealen Echokardiographie* zeigte sich eine deutlich exzentrische, hochgradige Mitralklappeninsuffizienz aufgrund eines partiellen Sehnenfadenabrisses im Segment P2 mit einem nach posterior gerichteten Jet (Abb. 1 und 2). Des Weiteren zeigten sich eine gute systolische linksventrikuläre Funktion und kein Hinweis auf einen Shunt auf Vorhofebene. Eine *echokardiographische Abklärung des rechten Ventrikels* ergab eine visuell leicht reduzierte Funktion bei einer reduzierten Anulusgeschwindigkeit von 7 cm/s, jedoch noch erhaltener longitudinaler rechtsventrikulärer Funktion („tricuspid annular plane systolic excursion“ 2 cm). Des Weiteren wurde eine mittelgradige Trikuspidalklappeninsuffizienz bei Anulusdilatation festgestellt.

**Figure 2:**
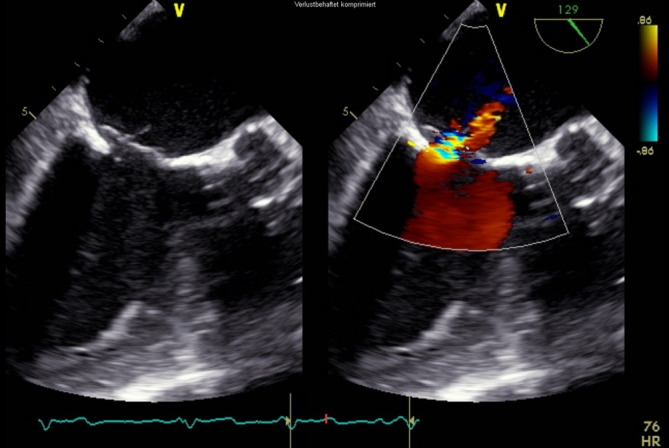

## Diagnose I

- Hochgradige Mitralklappeninsuffizienz aufgrund eines partiellen Sehnenfadenabrisses im Segment P2 mit einem nach posterior gerichteten Jet

## Therapie

**Figure 3:**
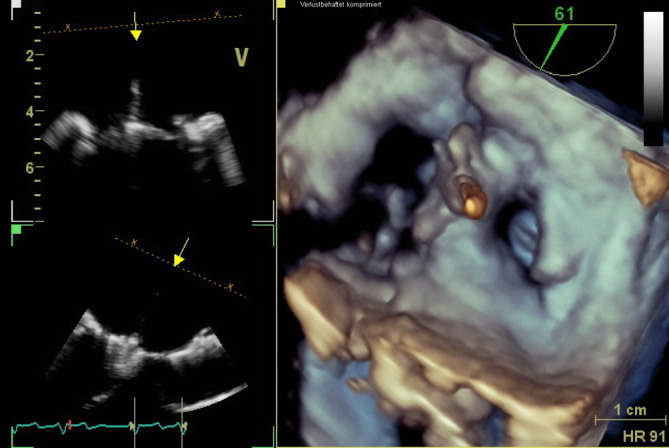

**Figure 4:**
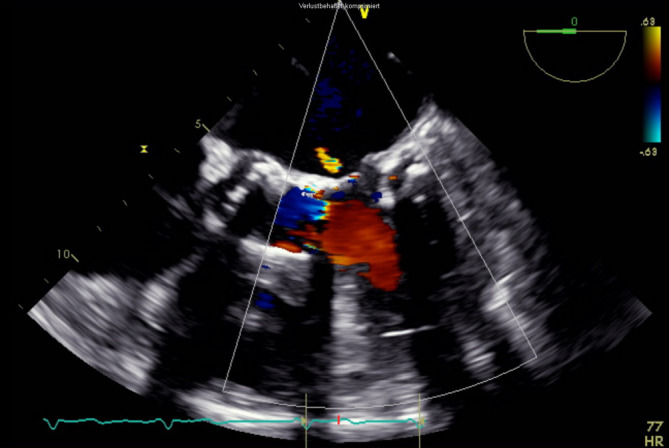

Die endovaskuläre Mitralklappenrekonstruktion wurde unter Vollnarkose begonnen. Der zentrale Venendruck zu Beginn des Eingriffs betrug 12 mm Hg bei einer Sauerstoffsättigung von 100 % und einem Blutdruck von 140/80 mm Hg. Es erfolgten die Punktion der rechten V. femoralis und Einlage einer 7 F-Schleuse mit anschließender transseptaler Punktion und Einlage einer PASCAL Guide Sheath (Edwards Lifesciences, Irvine, CA, USA). Es folgte die erfolgreiche Implantation eines PASCAL-Clips (Edwards Lifesciences) zwischen den Segmenten A2 und P2 (Abb. 3). Es zeigte sich eine adäquate Reduktion der Mitralklappeninsuffizienz nach Clipimplantation mit einer verbleibenden leicht- bis mittelgradigen Insuffizienz (Abb. 4). Die Untersuchung mit dem *Continuous-wave-Doppler* ergab keinen Hinweis auf eine relevante Stenosewirkung bei einem mittleren Druckgradienten von 4,36 mm Hg.

**Figure 5:**
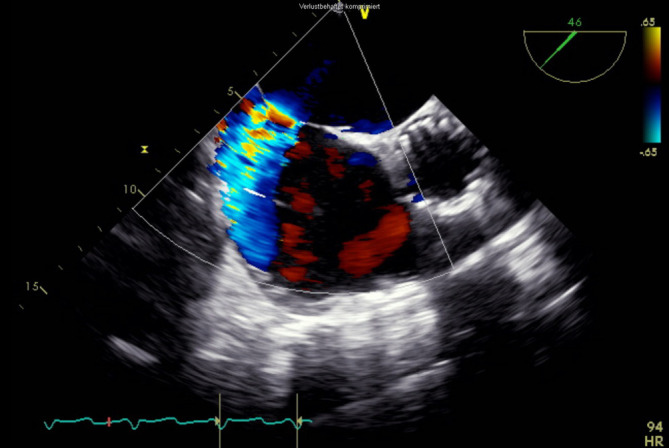

**Figure 6:**
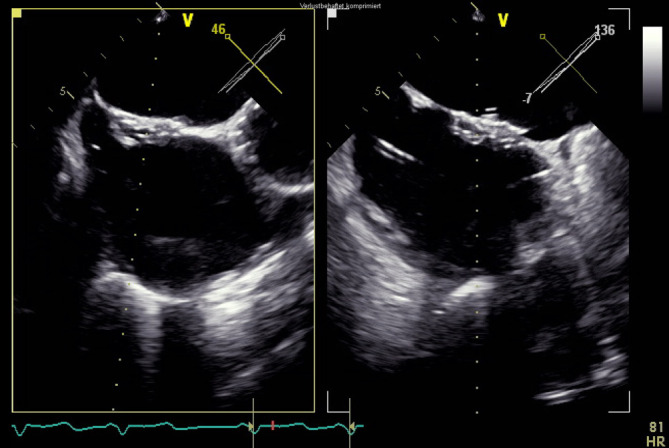

Nach Rückzug der transseptalen Schleuse kam es zu einem Anstieg des zentralen Venendrucks auf 20 mm Hg, einem Abfall des systolischen Blutdrucks auf 90 mm Hg sowie einem Abfall der Sauerstoffsättigung auf 80 %. In der *transösophagealen Echokardiographie* zeigte sich ein Atriumseptumdefekt mit einem relevanten Links-rechts-Shunt (Abb. 5). Es erfolgte der sofortige Verschluss mittels Vorhofseptumokkluder (GORE Septal Occluder 30 mm, Gore, Flagstaff, AZ, USA; Abb. 6). Hiernach zeigte sich kein Hinweis auf einen Restshunt auf Vorhofebene. Der zentrale Venendruck fiel auf 11 mm Hg, Blutdruck und Sauerstoffsättigung normalisierten sich.

## Diagnose II

- Iatrogener Atriumseptumdefekt mit relevantem Links-rechts-Shunt nach endovaskulärer Mitralklappenrekonstruktion

## Verlauf

Die Patientin wurde für 24 h auf unserer kardiologischen Intensivstation überwacht und konnte anschließend auf unsere Normalstation verlegt werden. Weitere *echokardiographische Kontrollen* ergaben eine bleibende Reduktion der Mitralklappeninsuffizienz sowie keinen erneuten Hinweis auf einen Atriumseptumdefekt. Die Patientin stellte sich zur Verlaufskontrolle 4 Wochen nach Intervention in unserer Ambulanz vor. Es zeigte sich eine deutliche Besserung der Belastungsdyspnoe.

## Diskussion

Iatrogene Atriumseptumdefekte als Folge der transseptalen Punktion im Rahmen einer endovaskulären Mitralklappenrekonstruktion treten häufig auf, allerdings meist ohne weitere Folgen für den Patienten. In seltenen Fällen können jedoch auch relevante klinische Langzeitschäden durch eine dauerhafte Rechtsherzbelastung oder aber akute Komplikationen auftreten [1]. Solche akuten Komplikationen können bedingt durch die Entfernung der transseptalen Schleuse auftreten und ein akutes Rechtsherzversagen auslösen, erkennbar an einer rechtsventrikulären Dilatation, einer Abnahme der longitudinalen rechtsventrikulären Funktion oder auch einem Abfall der Sauerstoffsättigung [2–5]. Bisherige Fallberichte konnten bereits den Nutzen eines endovaskulären Verschlusses iatrogener Atriumseptumdefekte nach Mitralklappenclipping zeigen [2, 3, 5].

Während einer Mitralklappenrekonstruktion ist ein ständiges hämodynamisches Monitoring wichtig

Unser Fallbeispiel zeigt die Wichtigkeit des ständigen hämodynamischen Monitorings während einer Mitralklappenrekonstruktion. Ein plötzlicher Anstieg des zentralen Venendrucks bzw. ein Abfall der rechtsventrikulären Funktion, der Sauerstoffsättigung oder des Blutdrucks sollte immer zur Abklärung eines möglichen Shunts auf Vorhofebene führen. Der sofortige Verschluss eines hämodynamisch relevanten Shunts mittels Okkluder-Device stellt eine sichere und schnelle Therapie dieser Komplikation dar.

## Fazit für die Praxis

- Ständiges hämodynamisches Monitoring während einer Mitralklappenrekonstruktion ist eine wichtige Überwachungsmaßnahme.
- Ein plötzlicher Anstieg des zentralen Venendrucks bzw. eine Abnahme der rechtsventrikulären Funktion oder der Sauerstoffsättigung sollte an einen Shunt auf Vorhofebene denken lassen.
- Der sofortige Verschluss mittels Okkluder-Device ist eine sichere und schnelle Therapieoption.

## References

[1] Schueler R, Öztürk C, Wedekind JA, Werner N, Stöckigt F, Mellert F, Nickenig G, Hammerstingl C (2015). Persistence of iatrogenic atrial septal defect after interventional mitral valve repair with the MitraClip system: a note of caution. JACC Cardiovasc Interv.

[2] Yeh L, Mashari A, Montealegre-Gallegos M, Mujica F, Jeganathan J, Mahmood F (2017). Immediate closure of iatrogenic ASD after Mitraclip procedure prompted by acute right ventricular dysfunction. J Cardiothorac Vasc Anesth.

[3] Al’Aref SJ, Bergman G, Wong SC (2016). Atrial septal defect closure for right-to-left shunting following a Mitraclip repair. J Invasive Cardiol.

[4] Losi MA, Strisciuglio T, Stabile E, Castellano G, de Amicis V, Saccenti A, Maresca G, Santoro C, Izzo R, Barbato E, Esposito G, Trimarco B, Rapacciuolo A (2015). Iatrogenic atrial septal defect (iASD) after MitraClip system delivery: The key role of PaO2/FiO2 ratio in guiding post-procedural iASD closure. Int J Cardiol.

[5] Huntgeburth M, Müller-Ehmsen J, Baldus S, Rudolph V (2013). Postinterventional iatrogenic atrial septal defect with hemodynamically relevant left-to-right and right-to-left shunt as a complication of successful percutaneous mitral valve repair with the MitraClip. Int J Cardiol.

